# Formation of Mitochondrial Outer Membrane Derived Protrusions and Vesicles in *Arabidopsis thaliana*

**DOI:** 10.1371/journal.pone.0146717

**Published:** 2016-01-11

**Authors:** Akihiro Yamashita, Masaru Fujimoto, Kenta Katayama, Shohei Yamaoka, Nobuhiro Tsutsumi, Shin-ichi Arimura

**Affiliations:** 1 Laboratory of Plant Molecular Genetics, Graduate School of Agricultural and Life Sciences, The University of Tokyo, Tokyo, Japan; 2 Laboratory of Plant Molecular Biology, Graduate School of Biostudies, Kyoto University, Kyoto, Japan; 3 PRESTO, Japan Science and Technology Agency, 4-1-8, Honcho, Kawaguchi, Saitama, 332-0012, Japan; Beijing Forestry University, CHINA

## Abstract

Mitochondria are dynamic organelles that have inner and outer membranes. In plants, the inner membrane has been well studied but relatively little is known about the outer membrane. Here we report that Arabidopsis cells have mitochondrial outer membrane-derived structures, some of which protrude from the main body of mitochondria (mitochondrial outer-membrane protrusions; MOPs), while others form vesicle-like structures without a matrix marker. The latter vesicle-like structures are similar to some mammalian MDVs (mitochondrial-derived vesicles). Live imaging demonstrated that a plant MDV budded off from the tip of a MOP. MDVs were also observed in the *drp3a drp3b* double mutant, indicating that they could be formed without the mitochondrial fission factors DRP3A and DRP3B. Double staining studies showed that the MDVs were not peroxisomes, endosomes, Golgi apparatus or trans-Golgi network (TGN). The numbers of MDVs and MOPs increased in senescent leaves and after dark treatment. Together, these results suggest that MDVs and MOPs are related to leaf senescence.

## Introduction

Mitochondria are double-membrane organelles in eukaryotic cells that are essential for energy production and supply, for the control of diverse metabolic pathways and for regulation of cell death [1, 2]. Because of their sessile lifestyle, plants must adapt to changing environmental conditions. This has resulted in plant mitochondria acquiring some unique metabolic attributes (e.g., alternative respiratory pathways) [3]. Since mitochondria originated through the endosymbiosis of an alphaproteobacterium and a eukaryotic host, the mitochondrial outer membrane is probably descendent from the outer membrane of the alphaproteobacteria and the endocytotic plasma membrane of the host [4]. The mitochondrial outer membrane mediates the import of proteins and lipids, the passage of small molecules and conduction of signaling events between the mitochondrion and the cytosol [5, 6].

Mitochondria are dynamic organelles that move around, change their shape and frequently undergo fusion and fission [7]. Mitochondrial fission is needed for maintenance of mitochondrial morphology [7, 8]. Plant mitochondria are typically more spherical than animal mitochondria and change their morphology depending on the life stage and growth conditions [9–12]. Dynamin-related proteins (DRPs) play essential roles in mitochondrial fission, because they polymerise and constrict mitochondria to facilitate their fission [8]. Two *Arabidopsis* DRPs (DRP3A and DRP3B) have been shown to be involved in mitochondrial fission [13–16], and in the *drp3a drp3b* double mutant, mitochondria are connected to each other, resulting in massive elongation [17]. Moreover, a plant-specific mitochondrial fission factor, ELONGATED MITOCHONDRIA1 (ELM1), localizes to the outer surface of mitochondria and interacts with DRP3A and DRP3B [18].

In previous studies of plant mitochondria, mitochondrial dynamics were usually studied by visualizing the inner membrane which was stained with a fluorescent dye such as MitoTracker or by visualizing fluorescently-labeled protein in the matrix [9, 10, 12, 17]. Therefore, the dynamic behaviors of the mitochondrial outer membrane were not visualized and have been somewhat neglected. For example, plant mitochondria were known to have tubular protrusions of the matrix, called matrixules [15], but it was uncertain whether the outer membrane exactly wrap the matrixule. Because the mitochondrial outer membrane is the outermost layer of mitochondria and also a strategic interface between the mitochondria and the cytosol and other organelles, it should also be observed to understand the dynamics of “whole” mitochondria. Recent investigations of the mitochondrial outer membrane in cultured human cells have demonstrated that mitochondrial proteins are sorted into mitochondrial-derived vesicles (MDVs) which are 70–100 nm in diameter and which are involved in intracellular transport and mitochondrial quality control [19, 20]. However, similar vesicles have not yet been documented in plant cells.

While investigating the behavior of GFP-labeled ELM1 during mitochondrial fission in high time resolution in Arabidopsis, we unexpectedly observed two structures of the mitochondrial outer membrane that might be related to animal MDVs and report them here. One is protrusions of the outer membrane (without the matrix marker) and the other is MDV-like structures budding off from the tips of these protrusions. Our subsequent investigations raise the possibility that these structures are related to dark-induced leaf senescence.

## Results

### Live imaging reveals the presence of MDV-like structures in *A*. *thaliana*

**Fig 1:**
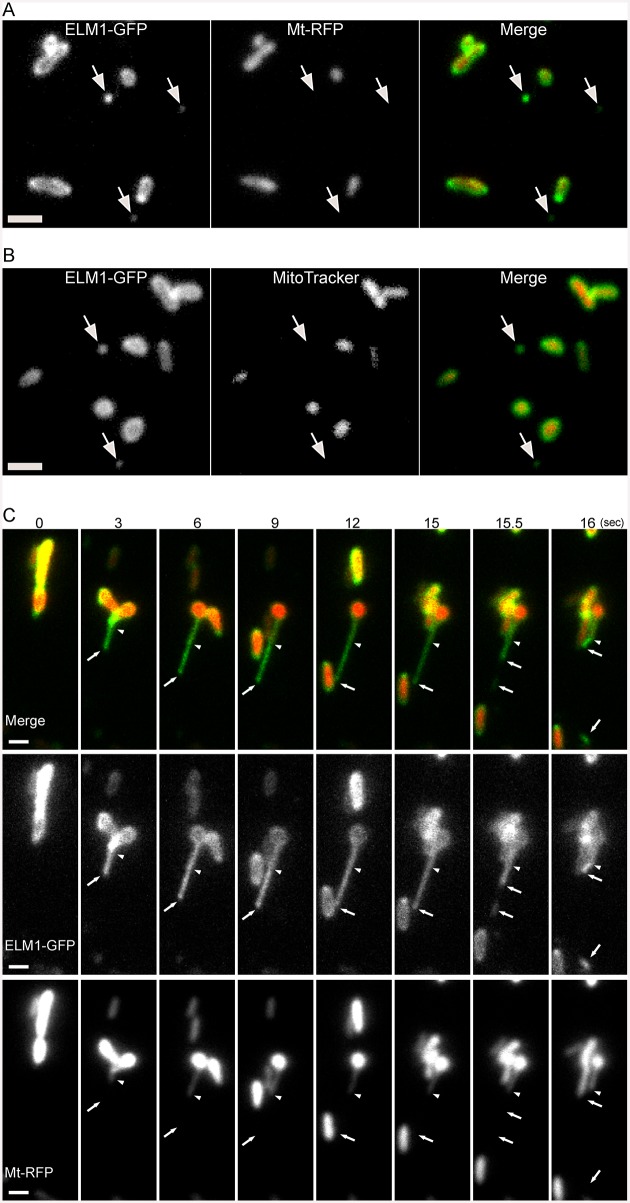
Microscopic observations of mitochondrial outer membrane, MDVs and MOPs. (A) Images of Arabidopsis leaf epidermal cells expressing both ELM1-GFP and Mt-RFP. Fluorescent fusions of ELM1 were expressed under the control of the ELM1 promoter. The arrows indicate MDVs. Bar = 1 μm. (B) Images of Arabidopsis leaf epidermal cells transfected with ELM1-GFP and stained by using a mitochondrial inner membrane marker, MitoTracker. Bar = 1 μm. The arrows indicate MDVs. (C) Time-course observation of the formation of a MDV. These are representative images from a movie (S1 Movie) that was recorded at 10 frames per second. The arrows indicate a MDV and the tip of a MOP, and the arrowheads indicate the tip of a protruding matrixule. The time stamp is in seconds. Bars = 1 μm.

To examine the distinctive morphology and dynamics of the mitochondrial outer membrane, we observed the outer membrane and the matrix simultaneously in epidermal cells of *Arabidopsis* cotyledons. In Fig 1A, the outer membrane was labeled with GFP fused with ELM1 under the control of its own promoter (ELM1-GFP) and the matrix was labeled with RFP fused to the mitochondrial matrix localization signal of the *Arabidopsis* mitochondrial F_1_-ATPase delta-prime subunit (Mt-RFP). As previously reported [5], almost all GFP fluorescence of mitochondrial outer membrane proteins was in the form of green oval-rings which were 0.5–1 μm in diameter and which surrounded the RFP fluorescence of the matrix (Fig 1A), indicating that ELM1-GFP was normally localized on the outer membrane. However, some of the GFP fluorescence was localized on small vesicle-like structures which were 50–200 nm in diameter and did not contain any RFP signal (Fig 1A, arrows). To determine whether the vesicle-like structures contained any of the inner membrane, we stained the leaves expressing ELM1-GFP with the mitochondrial inner membrane marker MitoTracker Orange. The MitoTracker signals were below the limit of detection (Fig 1B, arrows), indicating that little or no inner membrane was present.

In a time series presented in Fig 1C (S1 Movie), ELM1-GFP first protruded from Mt-RFP (Fig 1C, 0–9 sec). The protruding region remained for a few seconds (Fig 1C, 9–15 sec), and then was pinched off at the tip (Fig 1C, 15–16 sec). These results revealed that at least some of the small GFP-only structures (Fig 1C, arrows) were pinched off from the mitochondrial outer membrane protruding from the main body of mitochondria and the matrixule (Fig 1C, arrowheads). In these results, matrix marker Mt-RFP was below the limit of detection by fluorescence, indicating that these structures had little or no matrix. Then, we called the small vesicles “MDVs (Mitochondrial-Derived Vesicles)”, and named the protrusions of the outer membrane “MOPs (Mitochondrial Outer-membrane Protrusions)”.

### MDVs and MOPs are labeled with two other outer membrane proteins

**Fig 2:**
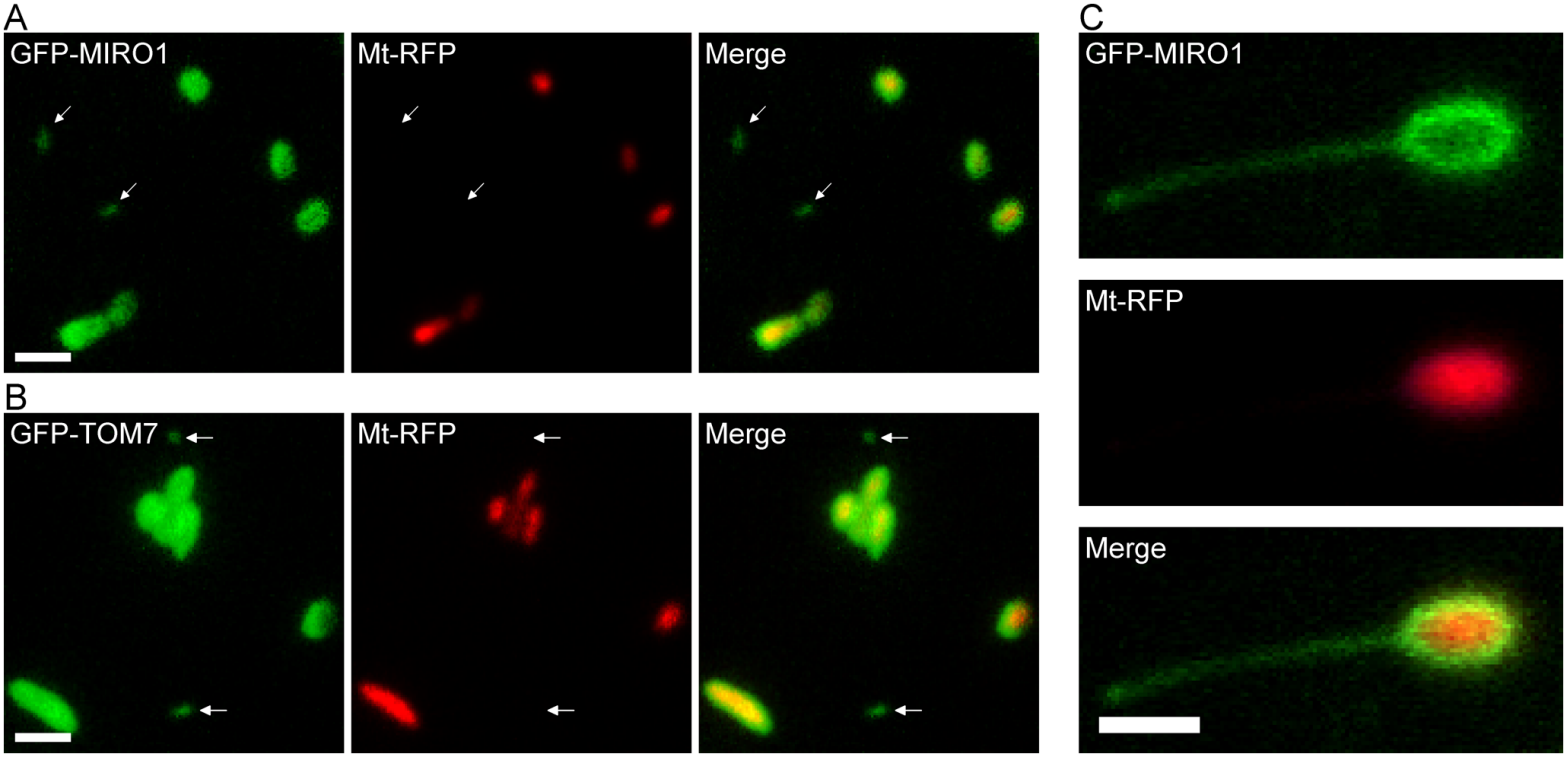
In vivo imaging of two GFP-labeled mitochondrial outer membrane proteins. (A) The images were obtained by VIAFM using leaf epidermal cells from 10-day-old transgenic Arabidopsis plants expressing GFP-labeled outer membrane proteins (MIRO1 and TOM7) and a matrix signal (Mt-RFP). (A) GFP-MIRO1. (B) GFP-TOM7. The arrows indicate MDVs. (C) A MOP labeled with GFP-MIRO1. Bars = 1 μm.

Next we examined whether MDVs and MOPs could also be visualized by labeling mitochondria with Mt-RFP and GFP-labeled outer membrane proteins, MIRO1 (mitochondrial *Rho* GTPase 1) [21] and TOM7 (translocase of the outer mitochondrial membrane subunit 7 kDa) [22]. The double staining showed objects (arrows in Fig 2A and 2B) that stained with MIRO1 and TOM7 but not with Mt-RFP. In addition, we observed a protrusion from a mitochondrion that was labeled with an outer membrane signal (MIRO1) but not with the matrix signal (Fig 2C, arrows). Taken together, these results indicate that MDVs and MOPs are not artifacts derived specifically from the expression of ELM1-GFP.

### MDVs don’t require mitochondrial fission factor DRP3

**Fig 3:**
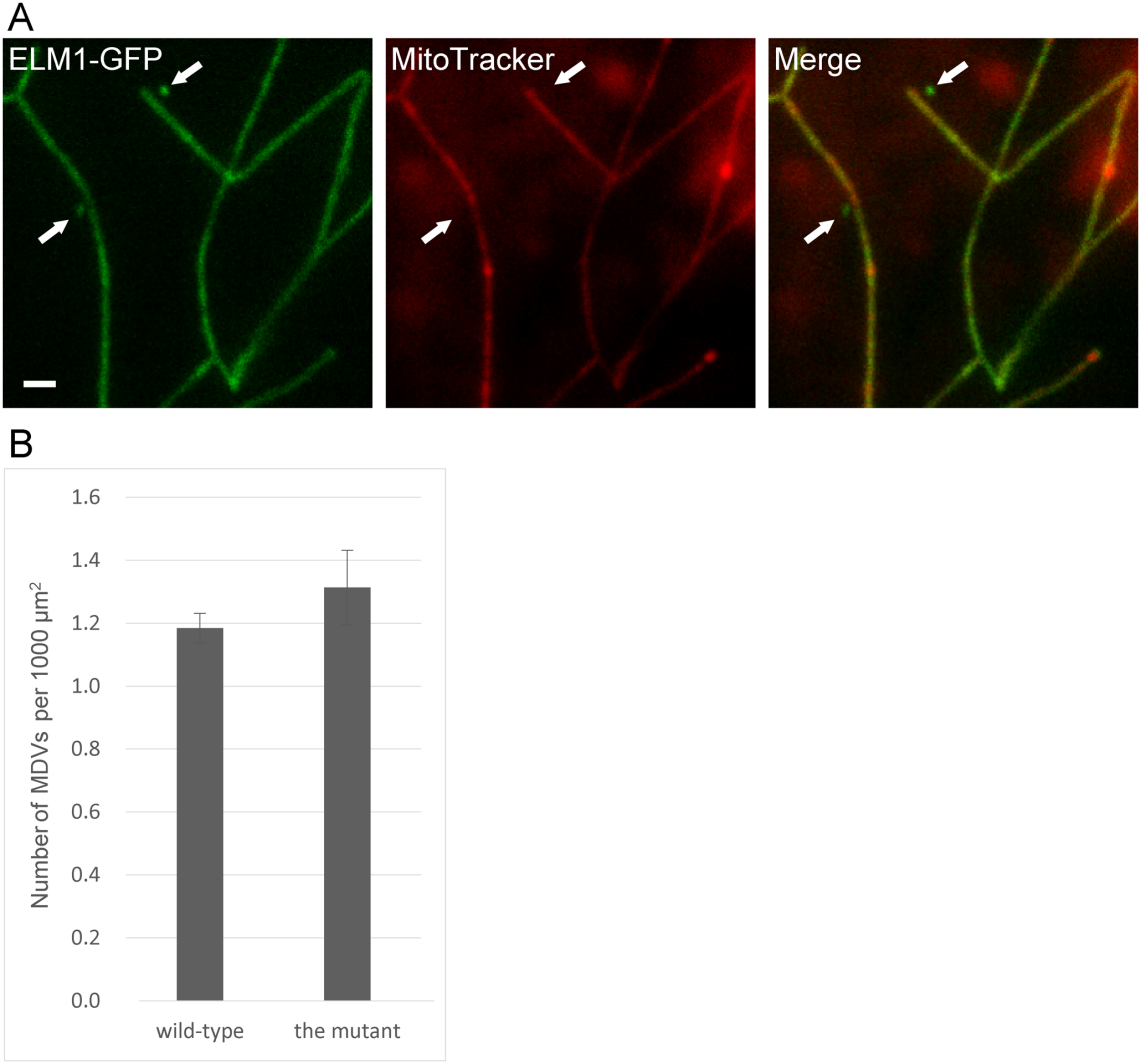
Microscopic observation of MDVs in the mitochondrial fission-defective mutant, *drp3a drp3b*. (A) The T-DNA insertion mutant, *drp3a drp3b*, transfected with ELM1-GFP was stained with MitoTracker. The arrows indicate MDVs. Bar = 1 μm. (B) The numbers of MDVs in the double mutant and in wild-type. Images of 20 epidermal cells were analyzed with three replicates. Error bars represent standard error (SE).

The finding that MDVs were pinched off from MOPs (Fig 1C) raised the possibility that their formation was dependent upon mitochondrial fission factors DRP3A and DRP3B. To determine whether this mechanism required DRP3, we observed both mitochondrial membranes in the double T-DNA insertion mutant, *drp3a drp3b*. In leaf epidermal cells of the *drp3a drp3b* double mutant, mitochondria were not only elongated but also networked (Fig 3A). The Fig also shows two MDVs (arrows), which are identified by their ELM1-GFP fluorescence and absence of MitoTracker fluorescence. The numbers of MDVs in the double mutant and wild-type visualized with ELM1-GFP and MitoTracker were not significantly different (Fig 3B), suggesting that MDVs could be formed without DRP3A and DRP3B.

### MDVs differ from other organelles

**Fig 4:**
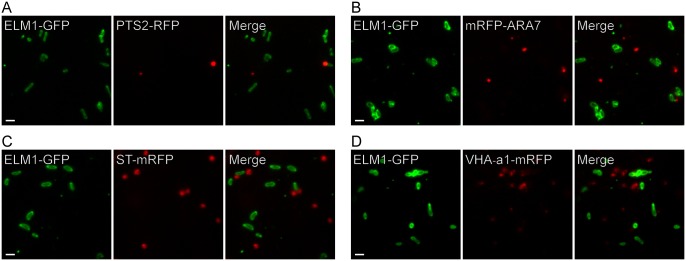
Visualization of MDVs and single-membrane organelles in living leaf epidermal cells of wild-type plants. *A*. *thaliana* transfected with ELM1-GFP were crossed with transgenic plants expressing (A) PTS2-RFP (Peroxisome Targeting Signal 2): Peroxisomes, (B) mRFP-ARA7 (*A*. *thaliana* Rab GTPase 7): Endosomes, (C) ST-mRFP (Syalyl Transferase): Golgi apparatus, and (D) VHA-a1-mRFP (Vacuolar H^+^-ATPase subunit a1): TGN. Bars = 1 μm.

ELM1-GFP did not colocalize with markers for four other single-membrane organelles (peroxisomes, endosomes, trans-cisternae of the Golgi apparatus and trans-Golgi network (TGN)) (Fig 4), demonstrating that MDVs are different from these organelles.

### Dark treatment increases the number of MDVs and MOPs in mature leaves

**Fig 5:**
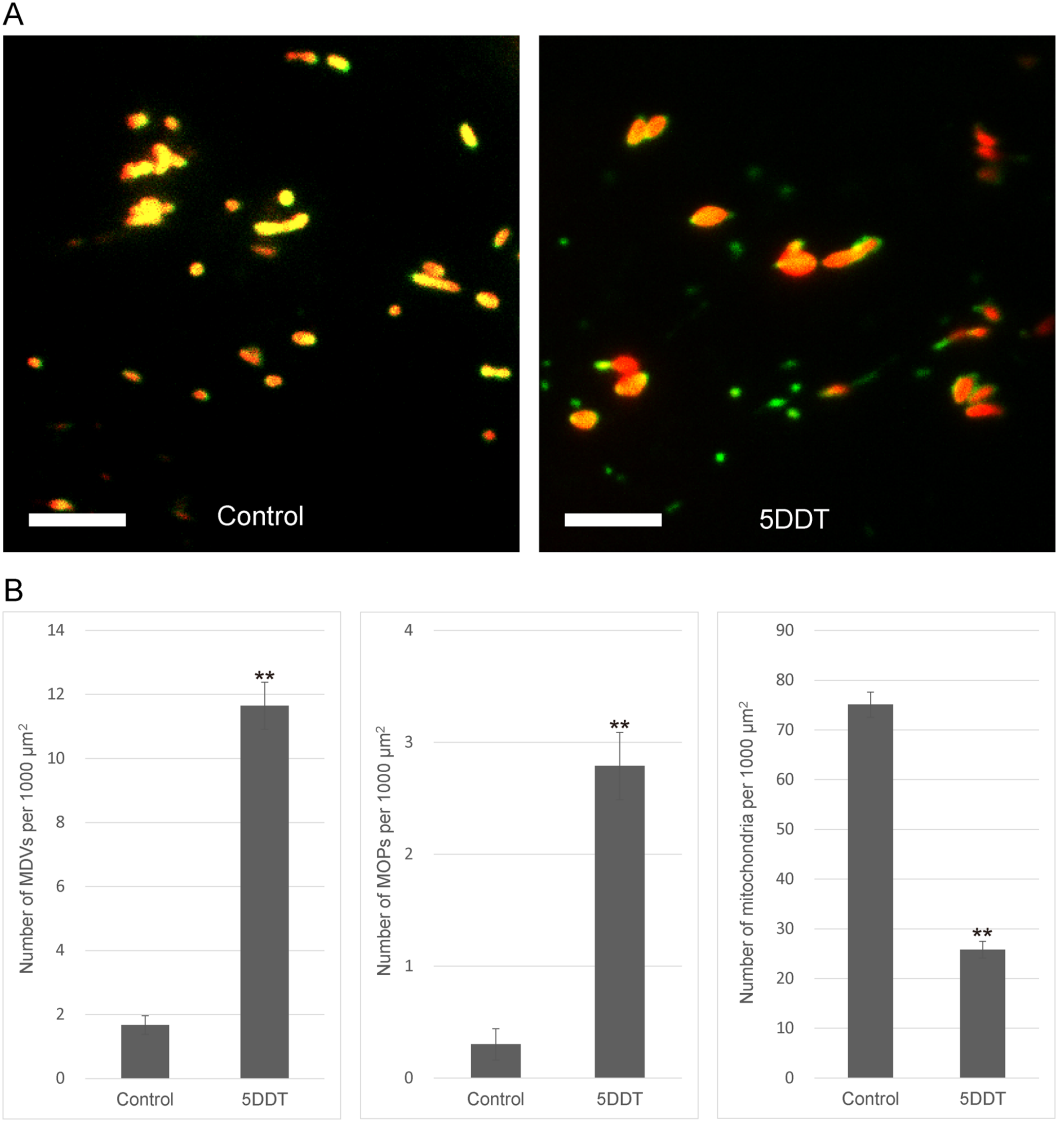
Effect of dark treatment on MDVs and MOPs. (A) Representative merged fluorescent images of epidermal cells in detached rosette leaves from 6-week-old transgenic plants expressing ELM1-GFP and Mt-RFP under normal light conditions (Control: left) and after 5-days of dark treatment (5DDT: right). MDVs and MOPs are indicated by green fluorescence only. Bars = 5 μm. (B) Statistical analyses of images of 30 epidermal cells like those shown in (A) with three replicates. Error bars represent standard error (SE). Asterisks indicate statistically significant differences between 5DDT and Control (Student’s *t*-test), **P<0.01.

During the course of this study, we noticed that there were more MDVs observed in early senescent leaves than in young leaves (S1 Fig). Subsequently, we counted the numbers of MDVs and MOPs in the detached leaves with light- or dark-treatment (Fig 5A). Surprisingly, when detached mature leaves were placed in the dark for 5 days, the numbers of MDVs and MOPs increased 6-fold and 9-fold, respectively (Fig 5B, left and center). At the same time, the number of mitochondria decreased by two-thirds (Fig 5B, right). The morphology of mitochondria did not elongate (Fig 5A), suggesting that the decrease was not due to mitochondrial fusion.

## Discussion

In this work, we describe two mitochondrial-derived structures that we call MDVs and MOPs. Previous studies of mitochondrial outer membrane proteins in plant cells showed images of MDV-like structures [5, 21], but these structures were not documented or further investigated. The structures that we observed had outer membrane signals but the matrix and inner membrane signals were below the limit of detection. To assure that MDVs were derived from the outer membrane and not the matrix, we had to observe both components simultaneously. Although most of the signals from the outer membrane markers were associated with signals from the matrix marker (indicating intact mitochondria), some of the signals appeared without the matrix signal or the inner membrane signal (indicating MDVs) (Fig 1A and 1B). Leaf epidermal cells had about one or two MDVs per 1000 μm^2^ (Fig 3B).

Moreover, we observed the budding off of a MDV from the tip of a MOP (Fig 1C). During the formation of a MDV, we observed not only a matrixule but also an extended outer membrane structure without the matrix marker (indicating MOPs) on the tip of the matrixule, as the two compartments of mitochondria were visualized simultaneously. Although MDVs and MOPs probably lacked the matrix marker and the inner membrane marker (Fig 1), these results do not exclude the possibility that those structures contain small molecules or proteins derived from the mitochondrial intermembrane space. Similar structures were observed when the outer membrane was labeled with two other outer membrane proteins (Fig 2), although it is unclear whether they are the same as the structures shown in Fig 1.

Although the formation of MDVs doesn’t require DRP3A and DRP3B (Fig 3), we cannot rule out the involvement of other DRPs, such as DRP5B, which participates in the fission of chloroplasts and peroxisomes without the DRP3 complex [23]. MDVs appear not to be other single-membrane organelles as they lack markers of peroxisomes, endosomes, Golgi apparatus and TGN (Fig 4).

Since the number of MDVs and MOPs increased after continuous dark treatment (Fig 5), an increase in the number of MOPs might lead to an increase in the number of MDVs. Placing individual leaves in the dark is known to lead to leaf senescence [24, 25]. Moreover, the finding that the number of mitochondria decreased sharply in the dark (Fig 5B) may be a result of leaf senescence as mitochondria are known to decrease during leaf senescence [12, 26], resulting in autophagic degradation of chloroplasts and mitochondria [27, 28]. Thus, it is presumed that the decrease of mitochondria was caused by the autophagic degradation of organelles during leaf senescence. However, it is possible that starvation caused by dark treatment also affected our results. Additionally, since chloroplast stroma without thylakoids can be partially mobilized to the vacuole by autophagy via spherical bodies named rubisco-containing bodies (RCBs), the generation of MDVs may also be related to mitochondrial autophagic processes during leaf senescence.

In mammalian cells, various types of MDVs have been reported [19, 20]. Some of them contain a matrix marker and others exclude the matrix marker [19]. In addition, some MDVs are transported to peroxisomes and others are transported to lysosomes where they were degraded [20]. The morphology of plant MDVs (Fig 1A and 1B) is similar to the morphology of some mammalian MDVs [19, 20], and neither of them requires DRPs for their formation (Fig 3). Although mammalian and plant MDVs are derived from mitochondrial membranes, their roles and destinies may be very different.

To the best of our knowledge, this is the first report of structures derived from the mitochondrial outer membrane in plant cells. In this paper, we observed MDVs excluding the matrix marker only; however there could be other types of plant MDVs. Future studies on MDVs and MOPs will highlight their precise functions and the formation mechanism of MDVs from the tips of MOPs.

## Materials and Methods

### Construction of plasmids

Plasmids expressing *ELM1*:*GFP* [18], *Mitochondria*:*RFP* [29], *GFP*:*MIRO1* [21] and *PTS2*:*RFP* [17] have been described previously. *GFP*:*TOM7* was constructed with Gateway cloning technology (Life Technologies). Open reading frame (ORF) of TOM7 subunit 2 was amplified by PCR, and was then cloned into pDONR207 Gateway entry vector via the Gateway BP reaction (Life Technologies). The resulting entry vector was used in LR reaction (Life Technologies) with the destination binary vector pK7WGF2 (Flanders Interuniversity Institute for Biotechnology, Belgium) to link GFP to the N-terminus of TOM7 [30]

### Plant materials and growth conditions

Plants (*A*. *thaliana*, ecotype Columbia-0) were grown on jiffy pellets (Jiffy Products) at 22°C under 16-h of diurnal light or continuous light (50–100 μmol m^-2^sec^-1^). T-DNA insertion mutants *drp3a drp3b* and transgenic plants expressing both ELM1-GFP and Mt-RFP or PTS2-RFP were generated as described previously [17, 18, 29]. Transgenic plants expressing GFP-TOM7 was generated by floral dipping with *Agrobacterium tumefaciens* strain C58C1 [31]. Transgenic plants expressing *mRFP*:*ARA7* [32], *ST*:*mRFP* [33] and *VHA-a1*:*mRFP* [34] were gifts from Dr. T. Ueda (The University of Tokyo), Dr. T. Uemura (The University of Tokyo) and Dr. K. Shoda (RIKEN). Transgenic plants that expressed combinations of GFP, RFP and mRFP fusions were generated by cross-pollination, and the F1 generations were used for microscopic observations.

### Microscopic observations

Microscopic observations were performed by using VIAFM (Variable incidence angle fluorescent microscopy) as described previously [35]. This technique makes it possible to observe both the outer membrane and the matrix simultaneously with millisecond temporal resolution because of extremely high signal-to-noise (S/N) ratio [35]. All images were acquired via a fluorescent microscope, Eclipse Ti (Nikon), with a cooled-CCD camera, Zyla 4.2 Scientific CMOS (Andor), controlled by NIS-Elements (Nikon). To make representative images, we selected appropriate images and assembled into panels using Photoshop CS6 (Adobe).

### MitoTracker orange staining

Small sections (5–30 mm^2^) from 4-week-old plants were cut out of the leaves with a sharp razor blade, stained with 1 μM MitoTracker Orange CMTMRos (Life Technologies) for about 30 minutes, and washed three times with distilled water.

### Quantitative analysis in the double mutant and in wild-type

4-week-old plants (the double mutant and wild-type) transfected with ELM1-GFP were cut out and stained with MitoTracker, and 20 epidermal pavement cells were observed and imaged with VIAFM in each experiment for three replicates. Cell areas were calculated with ImageJ bundle software (http://rsb.info.nih.gov/ij/). The MDVs were counted in each image and expressed as the average number per 1000 μm^2^.

### Dark treatment

Fully extended rosette leaves were detached at the middle of their petioles from 6-week-old plants and incubated on a stack of wet filter papers in a Petri dish. The Petri dishes were wrapped with either transparent film (control) or aluminum foil (5DDT), and kept in an incubator at at 22°C under a 16-h photoperiod (50–100 μmol m-2sec-1) for 5-days. After treatment, 30 epidermal pavement cells in a leaf blade of one leaf, about 10 mm from the cut, were observed and imaged with VIAFM in each experiment for three replicates. Cell areas were calculated with ImageJ. The MDVs, MOPs and mitochondria were counted in each image and expressed as the average number per 1000 μm^2^.

### Statistical analysis

Data are presented as mean and SE of the biological replicates. Differences were tested by unpaired, two-tailed T test. Significant differences by p<0.01 are indicated by two asterisks.

## Supporting Information

S1 Fig Microscopic observation of MDVs in early senescent leaves. Representative merged fluorescent images of epidermal cells in young leaves (A) and in early senescent leaves (B) from the same 6-week-old transgenic plant expressing ELM1-GFP and Mt-RFP. The arrows indicate MDVs. Bars = 5 μm.
(TIF)

S1 Movie Live imaging of protrusion of the mitochondrial outer membrane and the formation of a MDV. The leaf was analyzed by means of VIAFM. The movie was recorded at 10 frames per second and plays in real-time. Bar = 1 μm.
(MP4)
