# Supplementary figures and images for: Formation of Mitochondrial Outer Membrane Derived Protrusions and Vesicles in Arabidopsis thaliana
Source: PLoS One. 2016 Jan 11;11(1):e0146717. doi: 10.1371/journal.pone.0146717 (PMC4713473; doi:10.1371/journal.pone.0146717)

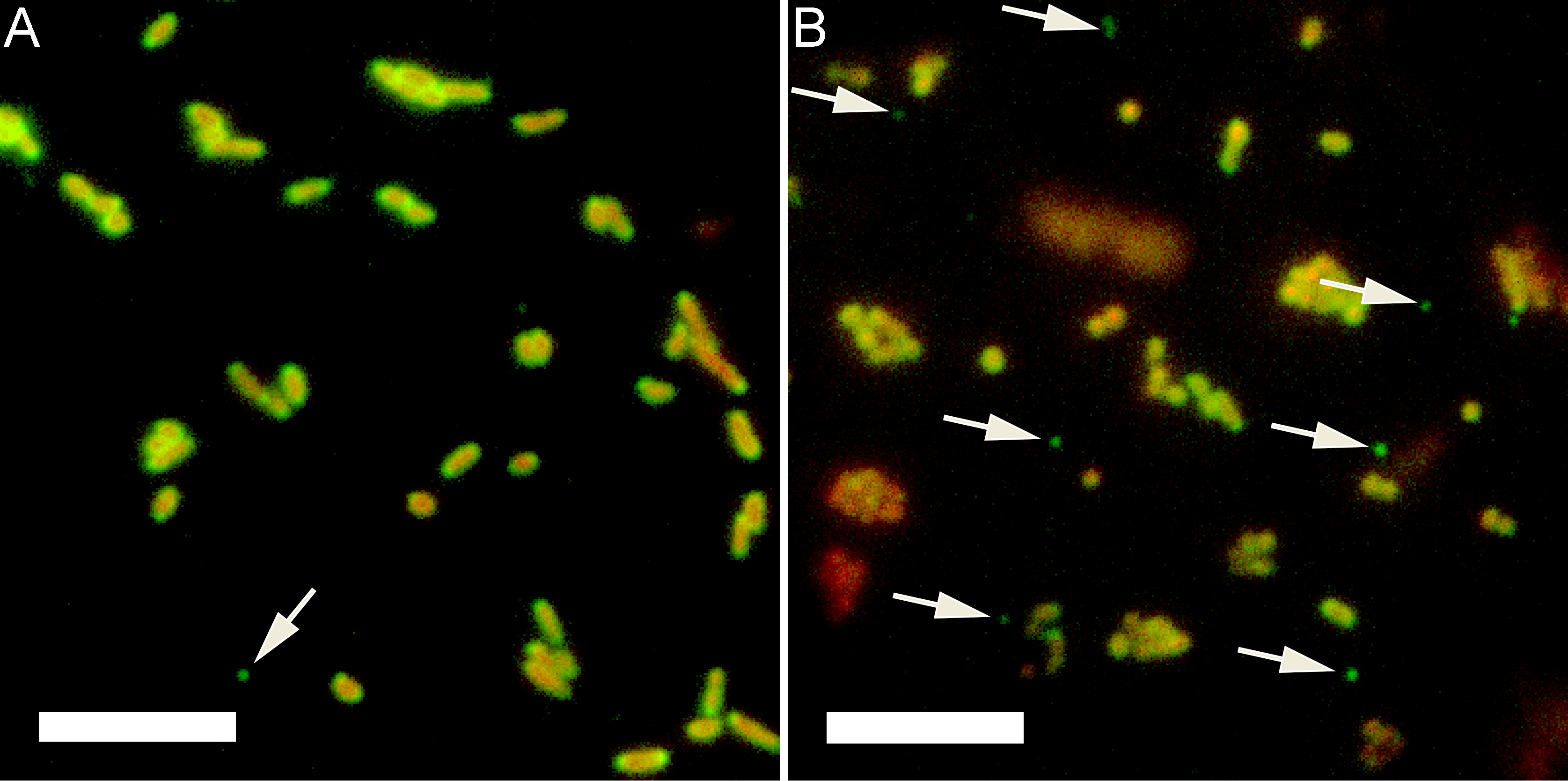

Supplement: S1 Fig — Representative merged fluorescent images of epidermal cells in young leaves (A) and in early senescent leaves (B) from the same 6-week-old transgenic plant expressing ELM1-GFP and Mt-RFP. The arrows indicate MDVs. Bars = 5 μm. (TIF) [file pone.0146717.s001.tif]
